# Gel-Free 3D Tumoroids with Stem Cell Properties Modeling Drug Resistance to Cisplatin and Imatinib in Metastatic Colorectal Cancer

**DOI:** 10.3390/cells10020344

**Published:** 2021-02-06

**Authors:** Chiharu Sogawa, Takanori Eguchi, Yuri Namba, Yuka Okusha, Eriko Aoyama, Kazumi Ohyama, Kuniaki Okamoto

**Affiliations:** 1 Department of Dental Pharmacology, Okayama University Graduate School of Medicine, Dentistry and Pharmaceutical Sciences, Okayama 700-8525, Japan; caoki@md.okayama-u.ac.jp (C.S.); pon48yol@s.okayama-u.ac.jp (Y.N.); yokusha@bidmc.harvard.edu (Y.O.); kohyama@md.okayama-u.ac.jp (K.O.); k-oka@okayama-u.ac.jp (K.O.); 2 Advanced Research Center for Oral and Craniofacial Sciences (ARCOCS), Okayama University Graduate School of Medicine, Dentistry and Pharmaceutical Sciences, Okayama 700-8525, Japan; eaoyama@md.okayama-u.ac.jp; 3 Department of Oral and Maxillofacial Radiology, Okayama University Graduate School of Medicine, Dentistry and Pharmaceutical Sciences, Okayama 700-8525, Japan; 4 Division of Molecular and Cellular Biology, Department of Radiation Oncology, Beth Israel Deaconess Medical Center, Harvard Medical School, Boston, MA 02115, USA

**Keywords:** gel-free 3D culture, tumoroid, cisplatin resistance, imatinib (gleevec), tyrosine kinase inhibitor (TKI), spheroid, metastatic colorectal cancer (mCRC), stem cells

## Abstract

Researchers have developed several three-dimensional (3D) culture systems, including spheroids, organoids, and tumoroids with increased properties of cancer stem cells (CSCs), also called cancer-initiating cells (CICs). Drug resistance is a crucial issue involving recurrence in cancer patients. Many studies on anti-cancer drugs have been reported using 2D culture systems, whereas 3D cultured tumoroids have many advantages for assessing drug sensitivity and resistance. Here, we aimed to investigate whether Cisplatin (a DNA crosslinker), Imatinib (a multiple tyrosine kinase inhibitor), and 5-Fluorouracil (5-FU: an antimetabolite) alter the tumoroid growth of metastatic colorectal cancer (mCRC). Gene expression signatures of highly metastatic aggregative CRC (LuM1 cells) vs. low-metastatic, non-aggregative CRC (Colon26 and NM11 cells) were analyzed using microarray. To establish a 3D culture-based multiplexing reporter assay system, LuM1 was stably transfected with the *Mmp9* promoter-driven ZsGreen fluorescence reporter gene, which was designated as LuM1/m9 cells and cultured in NanoCulture Plate^®^, a gel-free 3D culture device. LuM1 cells highly expressed mRNA encoding ABCG2 (a drug resistance pump, i.e., CSC/CIC marker), other CSC/CIC markers (DLL1, EpCAM, podoplanin, STAT3/5), pluripotent stem cell markers (Sox4/7, N-myc, GATA3, Nanog), and metastatic markers (MMPs, Integrins, EGFR), compared to the other two cell types. Hoechst efflux stem cell-like side population was increased in LuM1 (7.8%) compared with Colon26 (2.9%), both of which were markedly reduced by verapamil treatment, an ABCG2 inhibitor. Smaller cell aggregates of LuM1 were more sensitive to Cisplatin (at 10 μM), whereas larger tumoroids with increased ABCG2 expression were insensitive. Notably, Cisplatin (2 μM) and Imatinib (10 μM) at low concentrations significantly promoted tumoroid formation (cell aggregation) and increased *Mmp9* promoter activity in mCRC LuM1/m9, while not cytotoxic to them. On the other hand, 5-FU significantly inhibited tumoroid growth, although not completely. Thus, drug resistance in cancer with increased stem cell properties was modeled using the gel-free 3D cultured tumoroid system. The tumoroid culture is useful and easily accessible for the assessment of drug sensitivity and resistance.

## 1. Introduction

The methodology of cell culture and tissue culture is essential in biology and medicine. Researchers have developed and used several three-dimensional (3D) culture systems, including colony formation assay [1], spheroids [2,3], organoids [4,5], and tumoroids [6,7]. There are several advantages of 3D culture compared to 2D culture: (1) The 3D cultured cells, tissues, organoids, and tumoroids are morphologically more similar to 3D tissues, organs, or tumors in the bodies. Organs and tumors developed in 3D culture systems obtain 3D structures with intercellular adhesion in vitro [6,7]; (2) 2D culture systems force attachment of tissues/cells on plastic plates induces forced integrin expression required for the attachment. There is no forced attachment in 3D culture systems. Instead, intercellular adhesion is formed among cells to form organ-like or tumor-like structures in the 3D culture systems [7]; (3) Cell cycle and cell growth in 3D culture systems resemble those in organs, tissues, or tumors in the bodies. Cells proliferate rapidly in 2D culture, whereas cells grow more slowly in 3D culture [7]. Slow cycling cancer cells, dormant cancer cells at the G0 phase, and cancer stem cells (CSCs, also called cancer-initiating cells (CICs) or tumor-initiating cells (TICs)) can cause low chemosensitivity, drug resistance, and relapse [8,9,10,11]. These slow and dormant cells are reconstructed in 3D tumoroids, which are accessible by researchers. Dormant cells are not chemosensitive but can be resistant against cell-cycle-dependent chemotherapeutics such as mitotic inhibitors, including taxanes and vinca alkaloids, topoisomerase inhibitors, and antimetabolites, including methotrexate and 5-fluorouracil (5-FU).

(4) Gel-free 3D culture systems are especially useful for adding and analyzing substances such as growth factors, drugs [12,13], and extracellular vesicles (EVs) [6,14,15] in the culture supernatants. Gel-free 3D culture systems include (i) rotary device, (ii) hanging drop array, (iii) aqueous two-phase system with dextran phase and polyethylene glycerol phase [16], (iv) low attachment culture plates such as Ultra-Low Attachment (ULA) plate and NanoCulture Plate^®^ (NCP) [3,6,7,12]. The mogul field structure on the NCPs restricts cells to sprawl on the base and enables tumor cells to migrate from a scaffold to another scaffold more actively than cells cultured on the 2D plate. The increased migration and lesser attachment of cancer cells on the NCPs enable tumor cells to form 3D tumoroids [3,6,7]; (5) Organoids and tumoroids have been developed recently, more similar to organs and tumors, respectively, in the bodies than spheroids. In organoids and tumoroids, complicated structures of organs and tumors can be reconstructed in vitro. For example, the tumor microenvironment such as vasculatures, immune cells, fibroblastic stromal cells, and normal epithelial cells can be reconstructed in vitro within organoids and tumoroids [17,18,19]; (6) Gene expression signatures, molecular profiles, and secretory phenotypes are particular in 3D tumoroids according to their physical properties, e.g., moonlighting metalloproteinases (MMPs) [6], ATP-binding cassette (ABC) transporters including ABC-G1 and ABC-G2 [15], and intercellular adhesion molecules such as E-cadherin [7,14], stem cell markers such as EpCAM/CD326, CD44 variants and CD133 [7], and other factors potentially involving stemness such as ESRP1/2, MUC1, and Delta/Notch signal [7]. The molecular profiles of tumoroids involve the properties of CSCs [7]. Accumulating evidence shows that tumors often include a CSC/CIC population, a cellular fraction essential for drug resistance, recurrence, and metastatic potential [20,21,22]. We have shown that LuM1, a metastatic colorectal cancer (mCRC) cell line, expresses an array of CSC/CIC markers, including phosphorylated STAT3 signaling [12], activated β-catenin signaling [12,13], and EpCAM/CD326 [12]. Thus, the gel-free 3D culture system is accessible and useful for modeling tumors with stem cell properties.

ABC transporter G2 (ABCG2) is a drug resistance protein, also called a breast cancer resistance protein (BCRP), as well as a stem cell marker, including cancer stem cells [23,24,25,26]. To detect ABCG2-positive stem cells, side population (SP) analysis has been used in flow cytometry [27,28,29,30,31]. Cells overexpressing ABCG2 pump out Hoechst 33342 dye and are detectable as SP in flow cytometry. Verapamil, an ABC transporters’ inhibitor, can block ABCG2 and reduce the SP rate [21,32,33]. In the present study, to characterize the stem cell properties of mCRC, we performed gene screening overexpressed in the highly metastatic, aggregative CRC (LuM1) compared to low-metastatic, non-aggregative mCRC (Colon26/CT26 or NM11). Among many CSC/CIC markers, we found ABCG2 to be upregulated in the highly metastatic, aggregative LuM1 cells. Therefore, we aimed to examine whether the LuM1 cells’ population contained ABCG2-positive CSC-like SP cells using Hoechst 33342, verapamil, and flow cytometry.

Drug resistance is a crucial issue in cancer, which induces recurrence or relapse in cancer patients. Some chemoresistance has been overcome; however, recent studies clarified resistance against molecularly targeted therapeutics [34]. Of note, ABCG2 transports anti-cancer agents such as irinotecan, 7-ethyl-10-hydroxycamptothecin (SN-38), gefitinib, Imatinib, methotrexate, and mitoxantrone from cells [26]. Many studies on drug resistance have been performed in 2D culture systems, whereas we recently examined chemosensitivity and chemoresistance in the gel-free 3D cultured tumoroids [12,13]. For example, 5-FU, an antimetabolite inhibiting DNA replication, inhibited the tumoroid growth of mCRC, while this agent did not inhibit the metastatic metalloproteinase activity of mCRC cells [13]. On the other hand, cortisol, an anti-inflammatory steroid hormone agent, inhibited the metalloproteinase activity of the mCRC, whereas this agent did not inhibit but rather promoted tumoroid growth [13]. Moreover, using the gel-free 3D culture system, we identified repurposing drugs for cancer therapy: (1) artesunate, an anti-malaria drug, inhibits the tumoroid growth of mCRC [13]; (2) benztropine, an anti-Parkinson disease drug, inhibits tumor growth and the metastasis of mCRC [12].

However, chemosensitivity and chemoresistance are not fully investigated in 3D culture systems. DNA crosslinkers such as cisplatin and tyrosine kinase inhibitors (TKI) such as Imatinib (Gleevec) have not been well examined in 3D tumoroid culture systems. It has also not been well studied whether 5-FU, a cell-cycle-dependent antimetabolite, is effective in tumoroids that include slow cycling, dormant cancer cells. Thus, in the present study, we aimed to investigate whether Cisplatin, Imatinib, and 5-FU alter the tumoroid growth of metastatic cancer cells in the gel-free 3D culture system, which can model a new system for the analysis of chemoresistance and chemosensitivity.

## 2. Materials and Methods

### 2.1. Gel-Free 3D Culture

For gel-free 3D culture, we used LuM1 and LuM1/m9 cells. LuM1 is a rapidly metastatic, aggregative CRC cell line generated from the parental murine CRC cell line Colon26 (aka CT26) [13,35,36]. LuM1/m9 reporter cells were established by the stable transfection of a murine *Mmp9* promoter (588 bp)-driven ZsGreen reporter construct into LuM1 cells [12,13]. For a 3D culture, LuM1/m9 cells were cultured using 96-well NanoCulture Plate^®^ (NCP; MBL Life Science, Nagoya, Japan) in RPMI 1640 medium supplemented with a 2 or 10% FBS or mTeSR1 stem-cell medium (Stemcell Technologies, Vancouver, BC, Canada), as described previously [7,13].

For 2D culture, we used LuM1, Colon26, and NM11 cells. Cells were maintained in RPMI 1640 containing 10% FBS supplemented with penicillin, streptomycin, and amphotericin B.

### 2.2. Chemicals and Drugs

Cisplatin (cis-diamminedichloro-platinum(II): CDDP) and 5-FU were purchased from Wako (Osaka, Japan). Imatinib was purchased from Focus Biomolecules (Plymouth Meeting, PA, USA).

### 2.3. Drug Treatment

For the analysis of in vitro tumorigenesis and chemoresistance, cells were seeded at a concentration of 5 × 10^3^ or 1 × 10^4^ cells per well in a 96-well NCP and then pre-cultured in mTeSR1 for 1, 3, or 4 days. Cisplatin was added to the pre-formed tumoroids at 10 μM, and the tumoroids were cultured 24 h before the viability assay. LuM1/m9 cells were seeded at 5 × 10^3^ cells/well in a 96-well NCP and then cultured in 10% FBS-containing RPMI 1640 medium for 72 h with or without drugs (Cisplatin, Imatinib, or 5-FU) in the other experiments. After each drug’s treatment, the size and viability of cell aggregate/tumoroid were measured as described below.

For 2D culture, 5.0 × 10^3^ cells were seeded in a 96-well 2D culture plate in RPMI1640 supplemented with 10% FBS for 24 h, then drugs were added and cultured for 24 h.

### 2.4. 3D Tumoroid-Based Multiplex Reporter Assay

Tumoroids of LuM1/m9 cells were formed in NCP as described above and previously [7,12,13]. The fluorescent area of each tumoroid or cell aggregate were analyzed using the ArrayScan HCS System (ThermoFisher Scientific, Waltham, MA, USA). Regarding cell aggregates formed in mTeSR1 medium, fluorescent areas greater than 300 μm^2^ were considered as tumoroids. *Mmp9* promoter activity was evaluated by an average fluorescence intensity per μm^2^ of all cells in a well. Experiments were performed with 3 or 4 biological replicates.

### 2.5. Cell Viability Assay

The ATP content was quantified using a CellTiter-Glo^®^ (CTG) luminescent cell viability assay (Promega, Medison, WI, USA). Briefly, from the total 200 μL of the culture supplement, 150 μL was removed, and 50 μL of CTG solution was added to each well and then suspended. The plate was rocked for 2 min and incubated for 10 min at 37 °C. The luminescence was measured in a plate reader (Molecular Devices, San Jose, CA, USA).

### 2.6. Side Population Analysis

Cells cultured on a 10 cm dish were washed with warmed PBS (−) (Ca^2+^ free and Mg^2+^ free), detached using 5 mL of Accutase (Innovative Cell Technologies, San Diego, CA, USA), and neutralized with 5 mL of the serum-contained medium. Cells were collected by centrifugation at 250× *g* for 3 min and suspended in the serum-contained medium. Cell aggregates were removed by using a 35 μm cell strainer. The density of cells was adjusted to 10^6^ cells/mL by adding the serum-contained medium. Cells were incubated at 37 °C by using a water bath, mixed with Hoechst 33342 (ThermoFisher Scientific) at a final concentration of 5 μg/mL and/or verapamil (Sigma) at a final concentration of 30 μg/mL, and then incubated at 37 °C for 90 min under dark conditions with stirring every 20 min. Cells were then centrifuged at 250× *g* for 3 min and washed with ice-cold PBS (−) containing 2% FBS. The cell density was adjusted to 10^7^ cells/mL and mixed with propidium iodide (PI) at a final concentration of 0.5 to 1.0 μg/mL. The cell aggregates were removed using the cell strainer, and single cells were analyzed using a FACSCalibur (BD Biosciences, Franklin Lakes, NJ, USA) at the Central Research Laboratory, Okayama University Medical School. For the elaboration of SP analysis, we used a FlowJo™ software (BD Biosciences).

### 2.7. Microarray and Bioinformatics

Colon26, LuM1, and NM11 cells were cultured for 3 days in 2D culture plates. Total RNA was extracted using the AGPC (acid guanidinium thiocyanate-phenol-chloroform extraction) method with TRI reagent^®^ (Molecular Research Center, Cincinnati, OH, USA). cDNA was synthesized from 0.1 μg of total RNA using a Low Input Quick Amp Labeling Kit (Agilent Technologies, Santa Clara, CA, USA), then hybridized to probes of a SurePrint G3 Mouse GE 8x60K v.2 Microarray system (Agilent Technologies). Raw data were registered to the Gene Expression Omnibus (GEO) database repository; accession ID: GSE97166; Colon26, GSM2553008; LuM1, GSM2553009; NM11, GSM2553010. Heat maps were generated using GraphPad Prism 8 (La Jolla, CA, USA).

### 2.8. RT-qPCR

RT-qPCR was performed as described [12,15]. LuM1 cells were cultured in 2D or 3D conditions for 4 days, and total RNA was extracted using the AGPC method with TRI reagent^®^ (Molecular Research Center). cDNA was synthesized using ReverTra Ace (Toyobo, Osaka, Japan). Real-time PCR was carried out using iQ SYBR (BioRad). Primers for *Abcg2* and *Hprt1* (an internal control) were listed in the previous report [15]. Relative mRNA levels were quantified by the ΔΔCt method using the formula as follows: fold change = 2^−ΔΔCt^.

### 2.9. Statistical Analysis

Data were expressed as the means ± SD unless otherwise specified. Statistical significance was calculated using GraphPad Prism (La Jolla, CA, USA). Three or more mean values were compared using a one-way analysis of variance with the pairwise comparison by Turkey’s method, while the two were made with an unpaired Student’s *t*-test; *p* < 0.05 was considered to indicate statistical significance.

## 3. Results

### 3.1. Stem Cell Properties of the Aggregative, Metastatic CRC Cell Line LuM1

We characterized LuM1 as highly aggregative, metastatic cells, whereas Colon26 and NM11 as low-metastatic, non-aggregative cells [15,36]. To further characterize these cells, we examined the gene expression signatures of CSC/CIC markers, including ABCG2, EpCAM, DLL1 (coding Delta-like 1), STAT, MUC1, ALDH1, PDPN (coding Podoplanin), OSMR (coding oncostatin M receptor), and LGR5, ABC transporters, metastatic markers (MMPs, Integrings, and EGFR), and pluripotency markers (Myc, NANOG, SOXs, POU5F1/OCT4, GATA3, KLF4, and BMI1) among these cell types.

**Figure 1:**
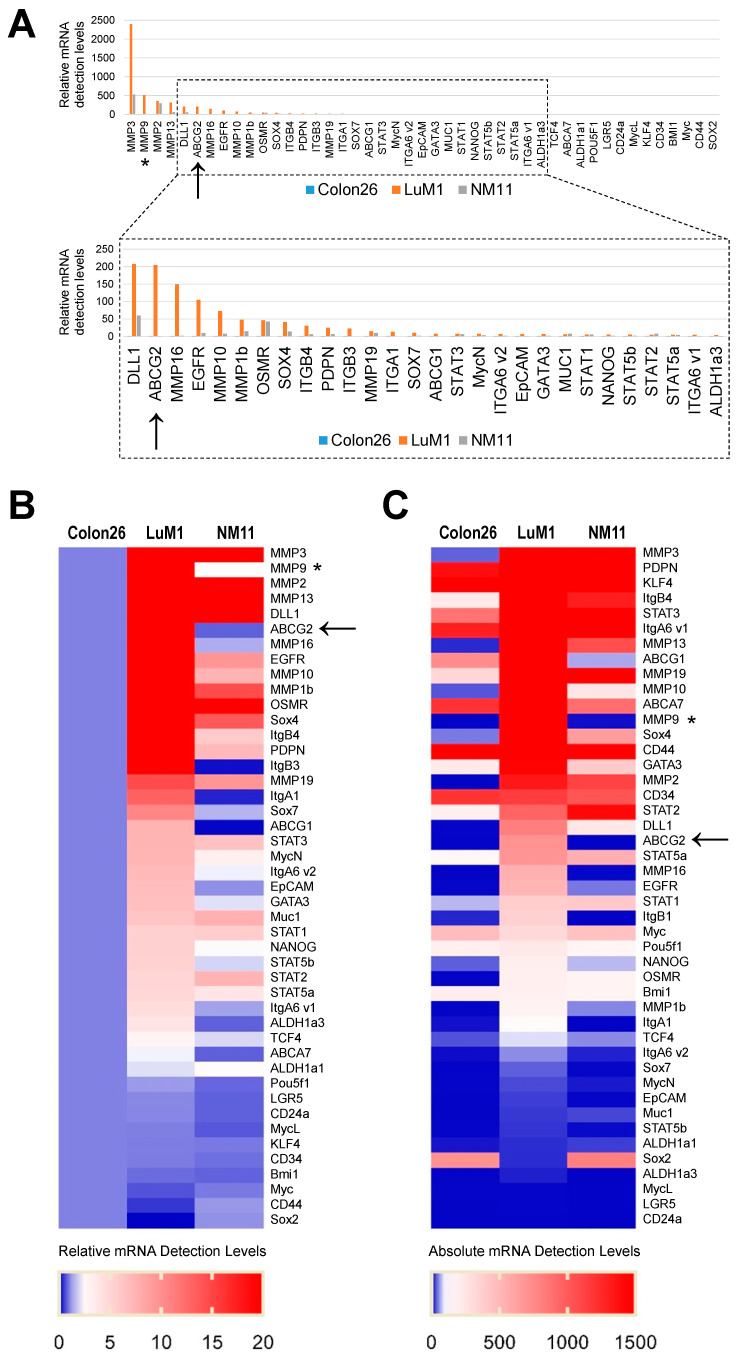
Gene expression signatures of cancer stem cell (CSC)/cancer-initiating cell (CIC) markers, metastatic markers, and drug resistance markers in highly and low-metastatic CRC cells. The mRNA levels in Colon26, LuM1, and NM11 were analyzed using microarray: (**A**) relative mRNA detection levels. The relative levels per the levels in Colon26 were shown; (**B**) heat map of the relative mRNA levels; (**C**) heat map of absolute mRNA levels. Arrows indicate ABCG2. Asterisks indicate MMP9.

The mRNAs increased in LuM1 than the other two cell lines were encoding CSC/CIC markers (ABCG2, DLL, EpCAM, podoplanin/PDPN, STAT3, STAT5, ALDH1a3, and TCF4), metastatic markers (Integrins β4, β3, α1, and α6, MMPs (MMP3, MMP9, MMP13, MMP2, MMP16, MMP1), and EGFR), pluripotent stem cell markers (SOX4, SOX7, N-Myc, NANOG, GATA3), and ABC transporters (ABC-G2, G1, A7) (Figure 1A–C).

These data indicated that the metastatic and stemness character of LuM1 involves the increased expression ABCG2, MMPs (including MMP9), and other multiple CSC/CIC marker genes.

### 3.2. Tumoroids Acquired Platinum-Resistance with ABCG2 Expression in Metastatic Colorectal Cancer Cells

**Figure 2:**
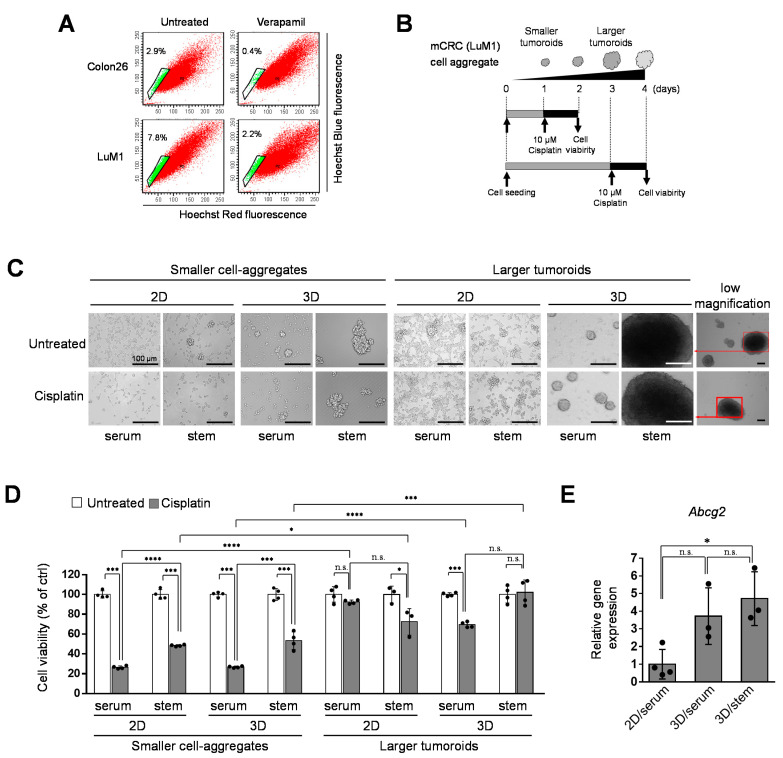
Tumoroids acquired platinum-resistance in metastatic colorectal cancer (mCRC) cells: (**A**) analysis of side population (SP) cells. Colon26 or LuM1 cells were treated with Hoechst 33342 and/or verapamil for flow cytometry. Green dots in the enclosed area indicate SP cells, whereas red dots are the main population cells; (**B**) schemes of the experimental protocol. Smaller cell aggregates were formed by pre-culturing LuM1 cells for 1 day. Larger tumoroids were formed by pre-culturing the cells for 3 days. Cisplatin at 10 μM was added to the tumoroids, and cell viability was measured; (**C**) representative images of cells and tumoroids cultured in 2D vs. 3D culture plates. Cells were cultured in a serum-containing medium or mTeSR1 stem cell medium. Scale bars, 100 μm; (**D**) cell viabilities of LuM1 cells and tumoroids treated with Cisplatin or untreated. *n* = 3 or 4 independent culture wells; (**E**) gene expression of *Abcg2* in 2D vs. 3D culture conditions. *n* = 3 or 4 independent culture wells. * *p* < 0.05, *** *p* < 0.001, **** *p* < 0.0001, n.s., not significant.

To ask whether ABCG2-expressing stem-like cells involve drug resistance in mCRCs, we then analyzed the side population (SP) in the LuM1 and Colon26. In the Hoechst SP technique, the cell-permeable DNA-binding dye Hoechst 33342 is loaded into the cell population of interest; stem cells, including CSCs, and early progenitors subsequently pump this dye out via an ABC pumps-dependent mechanism, resulting in a low-fluorescence tail (the SP) when the cells are analyzed by flow cytometry [27]. In the present study, we examined whether the mCRC cells’ population contains SP cells using Hoechst 33342 and flow cytometry. We first compared the rates of SP cells between the highly metastatic cells (LuM1) and the low-metastatic cells (Colon26) and then examined whether the rate of SP cells was reduced by verapamil, an ABCG2 inhibitor. The rate of SP cells in the LuM1 cells’ population was 7.8%, while that in Colon26 was lower (2.9%), suggesting that the ABCG2 positive SP rate was positively correlated with the metastatic potential of the cancer cell population (Figure 2A). Verapamil reduced the SP rate in the Colon26 population from 2.9 to 0.4% (Figure 2A). Additionally, verapamil reduced the SP rate in the LuM1 population from 7.8 to 2.2%.

We recently showed that 3D culture promoted cellular aggregates (tumoroid formation) more than 2D cultured cells and stemness enhancing medium (mTeSR1) promoted tumoroid formation more than serum-containing medium [7,13]. Then, we examined Cisplatin’s cytotoxicity to the smaller and larger tumoroids formed in 2D vs. 3D culture systems and different liquid environments (serum vs. stemness enhancing medium).

The NCP-based 3D culture condition appeared to promote tumoroid growth compared with a 2D culture system (Figure 2B,C), consistent with our previous data. Stemness enhancing medium (mTeSR1) also appeared to promote tumoroid growth compared to the serum-contained medium. Cisplatin reduced cell viability (ATP activity) in 2D culture conditions and in the serum-contained medium. In contrast, the cytotoxicity of Cisplatin was reduced by larger tumoroid formation based on the 3D culture system and the stemness-enhancing medium. Notably, the combination of the 3D culture and mTeSR1 markedly promoted cisplatin-resistant tumoroid growth (Figure 2D). Moreover, the combination of the gel-free 3D culture system and stemness-enhancing medium significantly increased ABCG2 gene expression (Figure 2E).

These data indicated that 3D-cultured tumoroids with cancer stem phenotype were more platinum-resistant than 2D-cultured cancer cells. Larger tumoroids were more platinum-resistant than smaller cell aggregates. The 3D tumoroid formation of mCSCs induced ABCG2 expression which may involve Cisplatin efflux.

### 3.3. Cisplatin Promoted Tumoroid Formation of Metastatic Colorectal Cancer

**Figure 3:**
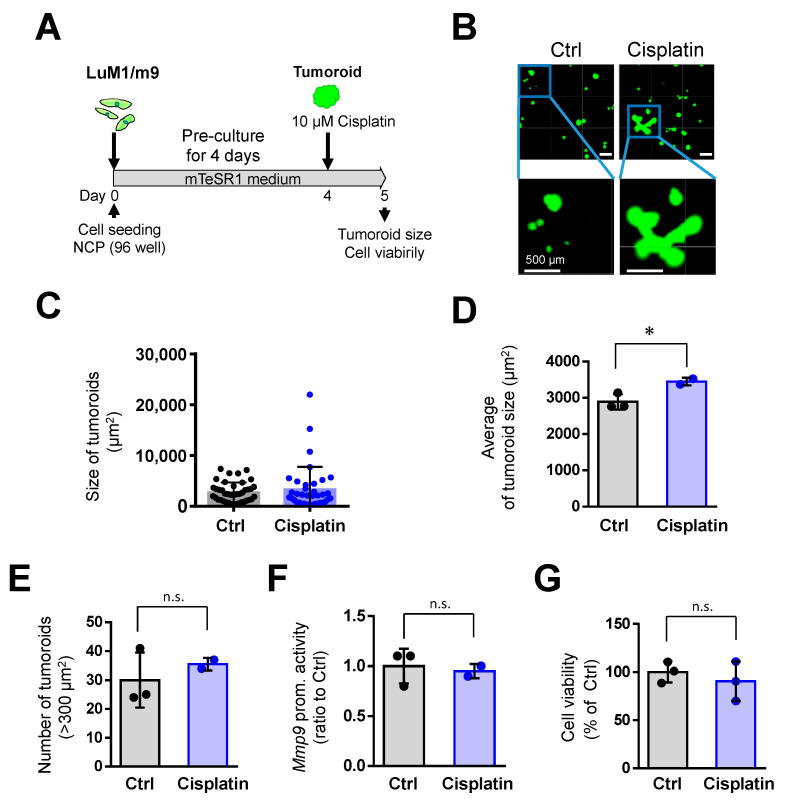
Cisplatin (at 10 μM) promoted the tumoroid growth of mCRC cells: (**A**) a scheme of the experimental protocol used for panel B–G. LuM1/m9 cells were pre-cultured for 4 days in mTeSR1 on NanoCulture Plate^®^ (NCP), and then Cisplatin at 10 μM was added; (**B**) representative images of tumoroids. Scale bars, 500 μm; (**C**) column scatters the plotting of tumoroid size. Representative data from a single well were shown; (**D**) average tumoroid size. *n* = 2 or 3 independent culture wells; (**E**) the number of tumoroids larger than 300 μm^2^. *n* = 2 or 3 independent culture wells; (**F**) *Mmp9* promoter activity. *n* = 3 independent culture wells; and (**G**) cell viability. *n* = 3 independent culture wells. * *p* < 0.05, n.s., not significant.

Then, we asked whether pre-formed, enlarged tumoroids were resistant to Cisplatin. Notably, Cisplatin tended to stimulate tumoroid growth (Figure 3A–C). The large tumoroids (10,000 to 20,000 µm^2^) were formed by cisplatin treatment, which was not found in the untreated group (Figure 3C). Cisplatin treatment significantly increased the tumoroid size as compared with the untreated group (Figure 3D). Cisplatin did not alter the rate of tumoroids larger than 300 µm^2^, viability, and Mmp9 promoter activity in the mCRC LuM1 cells (Figure 3E–G). These data indicated that the Cisplatin is ineffective in pre-formed, enlarged tumoroids, and rather promotes tumoroid growth.

**Figure 4:**
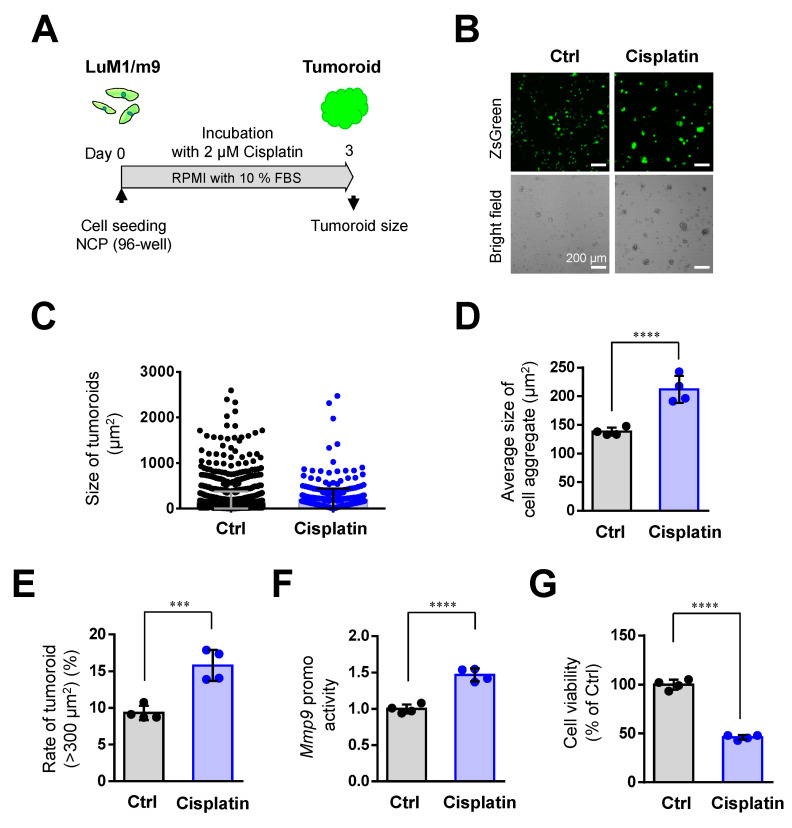
Cisplatin (at 2 μM) promoted the tumoroid formation of mCRC cells: (**A**) a scheme of the experimental protocol used for panel (**B**–**G**). LuM1/m9 cells were cultured with 2 μM Cisplatin for 3 days in a medium containing 10% FBS; (**B**) representative images of cell aggregates. Scale bars, 200 μm; (**C**) column scatters plotting of tumoroid size. Representative data from a single well were shown; (**D**) average tumoroid size. *n*= 4 independent culture wells; (**E**) the rate of tumoroids larger than 300 μm^2^. *n* = 4 independent culture wells; (**F**) *Mmp9* promoter activity. *n* = 4 independent culture wells; and (**G**) cell viability. *n* = 4 independent culture wells. *** *p* < 0.001, **** *p* < 0.0001.

Then, we examined whether a lower concentration (2 µM) of Cisplatin altered tumoroid formation. Cisplatin at 2 µM significantly increased tumoroid size (Figure 4A–D), the rate of large tumoroids > 300 µm^2^ (Figure 4E), and *Mmp9* promoter activity (Figure 4F). Meanwhile, Cisplatin at 2 µM lowered cell viability (Figure 4G).

We showed that mTeSR1, a stem cell-inducing medium promotes tumoroid formation more than the serum-containing medium in previous studies [6,7,15], as shown in Figure 2. Indeed, tumoroid formation was promoted by mTeSR1 more than serum-containing medium (compare Figure 4 vs. Figure S1). In the mTeSR1, Cisplatin at 2 µM did not increase tumoroid size, the rate of large tumoroids > 1000 µm^2^, and *Mmp9* promoter activity (Figure S1). Thus, it is conceivable that the tumoroid forming effect of Cisplatin was masked by the same effect of mTeSR1.

Nevertheless, these data indicated that Cisplatin at 2 µM can promote cell aggregation in mCRC cells.

### 3.4. Concentration-Dependent Effects of Imatinib on mCRC Cells in 2D Culture

**Figure 5:**
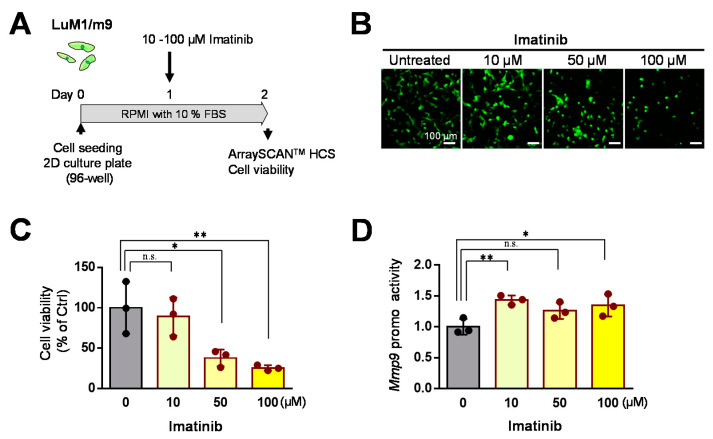
Effects of Imatinib on LuM1/m9 cells in the 2D culture system: (**A**) a scheme of the experimental protocol. Cells were treated with 10, 50, or 100 μM Imatinib or untreated; (**B**) representative fluorescence images of LuM1/m9 cells; (**C**) Mmp9 promoter activities. *n* = 3 independent culture wells; (**D**) cell viability. *n* = 3 independent culture wells. * *p* < 0.05, ** *p* < 0.01. n.s., not significant.

Then, we examined the effects of Imatinib at 10–100 µM on cell viability and *Mmp9* promoter activity in the 2D culture systems. Imatinib at 50 to 100 µM significantly lowered the cell viability in the 2D culture condition (Figure 5A–C). Imatinib at 10 to 100 µM increased *Mmp9* promoter activity in the 2D culture condition (Figure 5D).

These data indicate that Imatinib (at 50–100 µM) is cytotoxic to the mCRC cells in a concentration-dependent manner, whereas 10 µM Imatinib (at a low concentration) is ineffective on mCRC cells.

### 3.5. Imatinib Promoted Tumoroid Growth of mCRC

**Figure 6:**
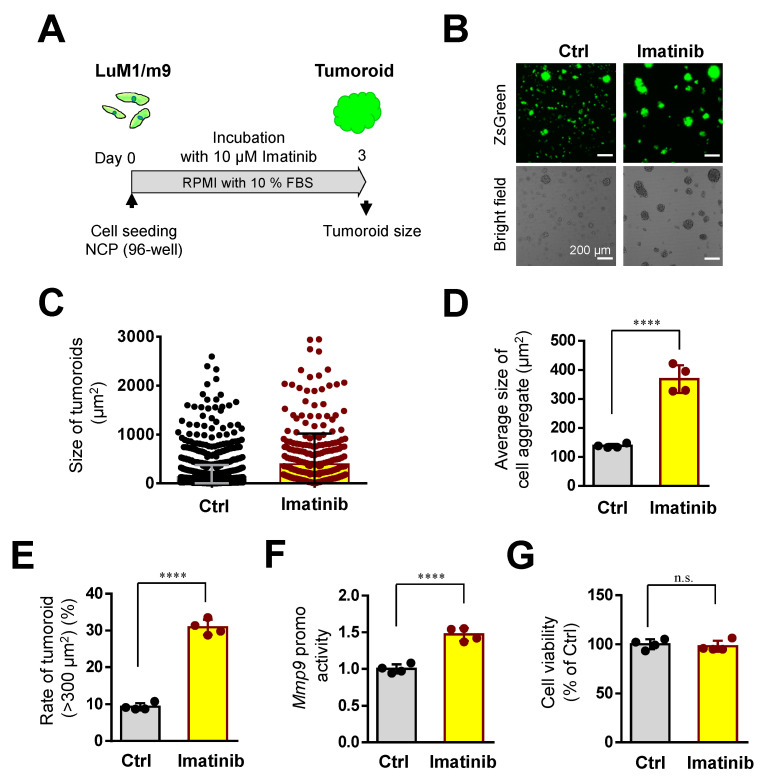
Imatinib promoted the tumoroid growth of mCRC: (**A**) a scheme of the experimental protocol used for panel (**B**–**G**). LuM1/m9 cells were cultured with 10 μM Imatinib for 3 days in a medium containing 10% FBS; (**B**) representative images of cell-aggregates with or without Imatinib. Scale bars, 200 μm; (**C**) column scatters plotting of tumoroid size. Representative data from a single well were shown; (**D**) the average size of cell aggregates or tumoroids. *n* = 4 independent culture wells; (**E**) the rate of tumoroids larger than 300 μm^2^. *n* = 4 independent culture wells; (**F**) *Mmp9* promoter activity. *n* = 4 independent culture wells; (**G**) and cell viability. *n* = 4 independent culture wells. **** *p* < 0.0001, n.s., not significant.

To examine whether Imatinib, a tyrosine kinase inhibitor, altered the tumoroid growth of mCRC cells, we used Imatinib at 10 µM in the following studies. Imatinib at 10 µM significantly promoted tumoroid growth (Figure 6A–E). The size of tumoroids was also significantly increased by imatinib treatment (Figure 6D). The rate of large tumoroids >300 µm^2^ was significantly increased by imatinib treatment (Figure 6E). The *Mmp9* promoter activity was significantly increased by imatinib treatment (Figure 6F). However, cellular viability was not altered by imatinib treatment (Figure 6G).

We then examined whether Imatinib altered tumoroid formation even in the tumoroid forming mTeSR1 medium. Imatinib at 10 µM did not alter tumoroid size, the rate of large tumoroids >1000 µm^2^, *Mmp9* promoter activity, or cell viability (Figure S2). Thus, it is conceivable that the tumoroid forming effect of Imatinib was masked by the same effect of mTeSR1.

These data indicated that Imatinib at a lower concentration (10 µM) can promote cell aggregation and the intercellular adhesion of mCRC cells.

### 3.6. 5-FU Inhibited Tumoroid Growth of mCRC, Although Not Completely

**Figure 7:**
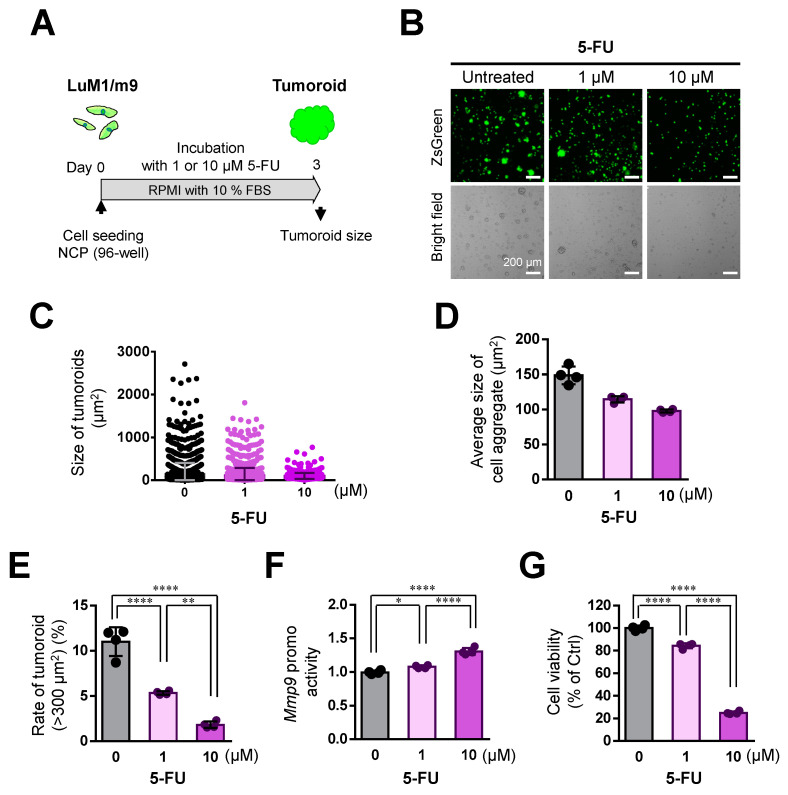
5-FU inhibited the tumoroid growth of mCRC, although not completely: (**A**) a scheme of the experimental protocol used for panel (**B**–**G**). LuM1/m9 cells were cultured with 5-FU at 0, 1, or 10 μM for 3 days in a medium containing 10% FBS; (**B**) representative images of cell aggregates of LuM1/m9. Scale bars, 200 μm^2^; (**C**) column scatters plotting of tumoroid size. Representative data from a single well were shown; (**D**) the average size of cell aggregates. *n* = 4 independent culture wells; (**E**) the rate of tumoroids larger than 300 μm^2^. *n* = 4 independent culture wells; (**F**) *Mmp9* promoter activity. *n* = 4 independent culture wells; and (**G**) cell viability. *n* = 4 independent culture wells. * *p* < 0.05, ** *p* < 0.01, **** *p* < 0.0001.

We then sought to examine whether 5-FU, an antimetabolite against DNA replication, altered the tumoroid growth of mCRC cells. 5-FU treatment significantly inhibited the tumoroid growth of mCRC LuM1 in a concentration-dependent manner (Figure 7A–E). 5-FU (1 or 10 µM) significantly reduced the size of tumoroids in a concentration-dependent manner (Figure 7B–D). 5-FU (1 or 10 µM) significantly reduced large tumoroids >300 µm^2^ in a concentration-dependent manner (Figure 7E). The *Mmp9* promoter activity in the survived cells was increased by 5-FU treatment (Figure 7F). 5-FU (1 or 10 µM) significantly reduced cell viability of mCRC tumoroids in a concentration-dependent manner (Figure 7G).

Then, we examined the cytotoxicity of 5-FU on the tumoroids in the mTeSR1 medium. 5-FU at 1 or 10 µM significantly inhibited tumoroid formation in a concentration-dependent manner in mTeSR1 medium (Figure S3A–E). Meanwhile, *Mmp9* promoter activity was increased by 5-FU treatment at 10 µM (Figure S3F). 5-FU (1 or 10 µM) significantly reduced the cell viability of mCRC tumoroids in a concentration-dependent manner (Figure S3G).

These data indicated that 5-FU is effectively cytotoxic to mCRC tumoroids, whereas survived 5-FU-resistant cells with increased MMP9 may involve relapse in mCRC.

## 4. Discussion

Drug resistance is an unsolved issue, especially in metastatic cancer, including mCRC. Many studies have examined chemoresistance in 2D culture systems, whereas our current study significantly demonstrated that the 3D culture system is a suitable, easily accessible tool to analyze drug resistance in metastatic cancer in vitro. Our study suggested that Cisplatin (a platinum-based DNA crosslinker) and Imatinib (TKI) were less effective and rather promoted the tumor growth of the metastatic CRC cells. On the other hand, 5-FU (an antimetabolite inhibiting DNA replication) more effectively combated the tumor growth of mCRC, although MMP9 positive metastatic cancer cells remained even after the 5-FU treatment, which was thought to be insensitive, dormant cancer cells in the tumoroids. Thus, the 3D tumoroid culture system is useful for analyzing drug resistance and metastatic cancer cells’ sensitivity.

Our study touched upon the mechanisms of platinum resistance using the gel-free 3D tumoroid model. Tumoroid growth induces both ABCG2 expression and exosome secretion [6,7,15]. Therefore, it is conceivable that Cisplatin could be secreted from cancer cells via ABC transporters such as ABCG2 efflux pump and/or exosomes (Figure S4). Indeed, several studies showed that Cisplatin was secreted with exosomes from cancer cells, defined as “resistance-associated secretory phenotype (RASP)” [34,37,38]. Moreover, cancer cell-derived EVs, including exosomes, promote the tumor growth of mCRC in vitro and in vivo [6,39]. Therefore, Cisplatin-induced exosomes may promote tumor growth, which may be a mechanism by which Cisplatin promoted tumor growth.

Our study also suggested that the ABCG2-mediated drug resistance involves 3D tumor formation with increased CSC/CIS properties. The aggregative, metastatic mCRC cells coordinately overexpressed mRNA encoding ABCG2, as well as other multiple CSC markers (Delta-like 1, oncostatin M receptor, podoplanin, STAT, MUC1), pluripotent stem cell markers (Sox4, Sox7, N-myc, GATA3, Nanog, Pou5f1/Oct4, KLF4), general stem cell markers (ALDH1, TCF4) and metastatic markers (MMPs, Integrins, EGFR). Consistently, we recently reported that tumoroid formation in metastatic prostate cancer cells PC-3 induced the expression of multiple stem cell markers [7]. Interestingly, Xie et al. reported that overexpressing microRNA-34a overcame ABCG2-mediated drug resistance to 5-FU in SP cells from colon cancer via suppressing DLL1 [29]. Another study showed the efficacy of atovaquone on EpCAM^+^CD44^+^ HCT-116 human colon cancer stem cells under hypoxia [40]. In this study, the EpCAM^+^CD44^+^ CSC also expressed increased mRNA levels of pluripotency genes such as Nanog, Sox2, Oct4 (aka. Pou5f1), and cMyc. Moreover, Atkinson et al. reported that EGFR and Prion protein promoted signaling via FOXO3a-KLF5 resulting in clinical resistance to platinum agents in CRC [41]. These data indicate that the drug resistance and recurrence in cancer may involve increased CSC properties. Additionally, we showed that CD44 variants were a more appropriate CSC marker, whereas CD44 standard (CD44s) was not [7]. Nevertheless, our study indicates that the ABCG2-mediated drug resistance involves 3D tumor formation with increased CSC/CIS properties.

Additionally, metastatic markers (MMPs, Integrins, and EGFR) were found in the list of increased mRNAs in the aggregative and metastatic cells, LuM1. MMPs (such as MMP3, MMP9, MMP13, MMP16, MMP10, MMP1b, and MMP19) were markedly increased at mRNA levels in the LuM1 compared with stem cell markers. We recently showed that MMP3 in extracellular vesicles, including exosomes, can promote tumor growth [6,39]. In these studies, we showed that the pro-tumorigenic effect of MMP3 involved the intra-nuclear translocation of MMP3, recently re-defined as “moonlighting metalloproteinase” [36,42,43,44,45]. Additionally, we found MMP9 in exosomes secreted by LuM1 cells (unpublished data). Therefore, *Mmp9* promoter activities measured in the present study could be a potential indicator of the MMP9-exosome secretion. We are currently studying exosome heterogeneity and its roles in tumorigenesis.

Our data showed that Imatinib at a low concentration was not cytotoxic but promoted the tumoroid growth of mCRC. Imatinib is a TKI that can inhibit multiple TKs, including receptor tyrosine kinases (RTK), in cancer cells. c-Kit is one of the RTKs targeted by Imatinib. Chau et al. reported that c-Kit mediated chemoresistance and tumor-initiating capacity of ovarian cancer cells through activation of Wnt/β-catenin-ABCG2 signaling [46]. Moreover, Imatinib was reported to upregulate compensatory integrin signaling in a mouse model of the gastrointestinal stromal tumor (GIST) [47]. It was also shown that Src family kinase-induced Imatinib resistance in a kinase-dependent manner in chronic myelogenous leukemia (CML) cells [48]. Src family kinase has been shown to mediate growth factor signals to multiple pathways, including STAT, Ras–MEK–ERK, and PI3K–Akt [47,49]. Among these pathways, STAT3 is crucial for activating the *MMP9* gene and cancer stem phenotype in the 3D tumoroid model [12] (Figure S4).

Moreover, Liu et al. reported exosome release in Imatinib-resistant human CML cells [50]. It has also been reported that ABCG2 transports anti-cancer agents such as Imatinib from cells [26]. Therefore, it was suggested that the integrins–Src–STAT3 signaling pathway, exosome release, and/or efflux via ABCG2 may cause Imatinib resistance. Another issue is that tumoroid formation is associated with the inhibition of epithelial-to-mesenchymal transition (EMT) [3]. EMT involves the migratory, invasive, and metastatic ability of cancer cells [14,51], and thus the inhibition of EMT is a potent strategy for cancer therapy. Tumoroid formation with intercellular adhesion is a result of an anti-EMT effect of the drugs. Imatinib may inhibit EMT and the invasive, metastatic ability of cancer cells. On the other hand, Perone et al. evaluated the resistant breast cancer spheroids for in vitro pro-invasive behavior [52].

Nevertheless, our multiplex system can evaluate both tumoroid formation and invasive activity measured by *Mmp9* promoter activity. In our current study, The *Mmp9* promoter activity was increased by Cisplatin, Imatinib, and 5-FU. Therefore, tumoroid forming activity and invasive activity may be independent issues. Anti-cancer drugs such as Cisplatin, Imatinib, and 5-FU can partially kill cancer cells in tumors while simultaneously stimulating the invasive activity of the remaining resistant cancer cells.

It has been known that many chemotherapeutics unselectively kill proliferating cells, including cancer cells and myeloid cells, in patients and thus often cause adverse effects, including myelosuppression. These limitations in chemotherapies have been overcome by molecularly targeted drugs, including antibody-based drugs. Moreover, precision medicine has enabled precise diagnosis-based medications, e.g., if the mCRC was EGFR-positive and RAS-wildtype, anti-EGFR antibody drugs such as cetuximab would be effective for cancer. In the present study, we showed that the gel-free 3D tumoroid assay is markedly useful to evaluate chemotherapeutics’ drug efficacy, while presumably that of antibody-based drugs as well. To study the efficacies of antibody-based drugs, it is recommended to add effector immune cells such as killer T cells, natural killer cells, or macrophages that express Fc receptors and can exert antibody-dependent cellular cytotoxicity (ADCC) or antibody-dependent cellular phagocytosis (ADCP) [34,53,54]. Nevertheless, it was conceived that large tumoroids might be resistant to ADCC and ADCP. We are currently exploring tumoroid-based tumor immunology. 

## 5. Conclusions

In conclusion, platinum resistance and imatinib resistance in metastatic colorectal cancer were modeled using the gel-free 3D cultured tumoroid with increased stem cell properties. The 3D tumoroid system is useful and easily accessible for drug assessment, including chemosensitivity and chemoresistance.

## Data Availability

Raw data of microarray were available at GEO database repository; accession ID: GSE97166 (all data), GSM2553008 (Colon26), GSM2553009 (LuM1), and GSM2553010 (LM11).

## Supplementary Materials

The following are available online at https://www.mdpi.com/2073-4409/10/2/344/s1, Figure S1. Effects of Cisplatin (at 2 μM) on tumoroid formation in mTeSR1 medium; Figure S2. Imatinib (at 10 μM) did not alter tumoroid formation in mTeSR1 medium; Figure S3. 5-FU inhibited the tumoroid growth of mCRC in mTeSR1 medium, although not completely; Figure S4: Data interpretation and perspective.
