# Supplementary material for: Gel-Free 3D Tumoroids with Stem Cell Properties Modeling Drug Resistance to Cisplatin and Imatinib in Metastatic Colorectal Cancer
Source: Cells. 2021 Feb 6;10(2):344. doi: 10.3390/cells10020344 (PMC7914642; doi:10.3390/cells10020344)
Supplement: Supplementary file 1 [file cells-10-00344-s001.pdf]

Supplementary Materials: Gel-free 3D Tumoroids with Stem Cell Properties Modeling Drug Resistance to Cisplatin and Imatinib in Metastatic Colorectal Cancer

Chiharu Sogawa, Takanori Eguchi, Yuri Namba, Yuka Okusha, Eriko Aoyama, Kazumi Ohyama, and Kuniaki Okamoto

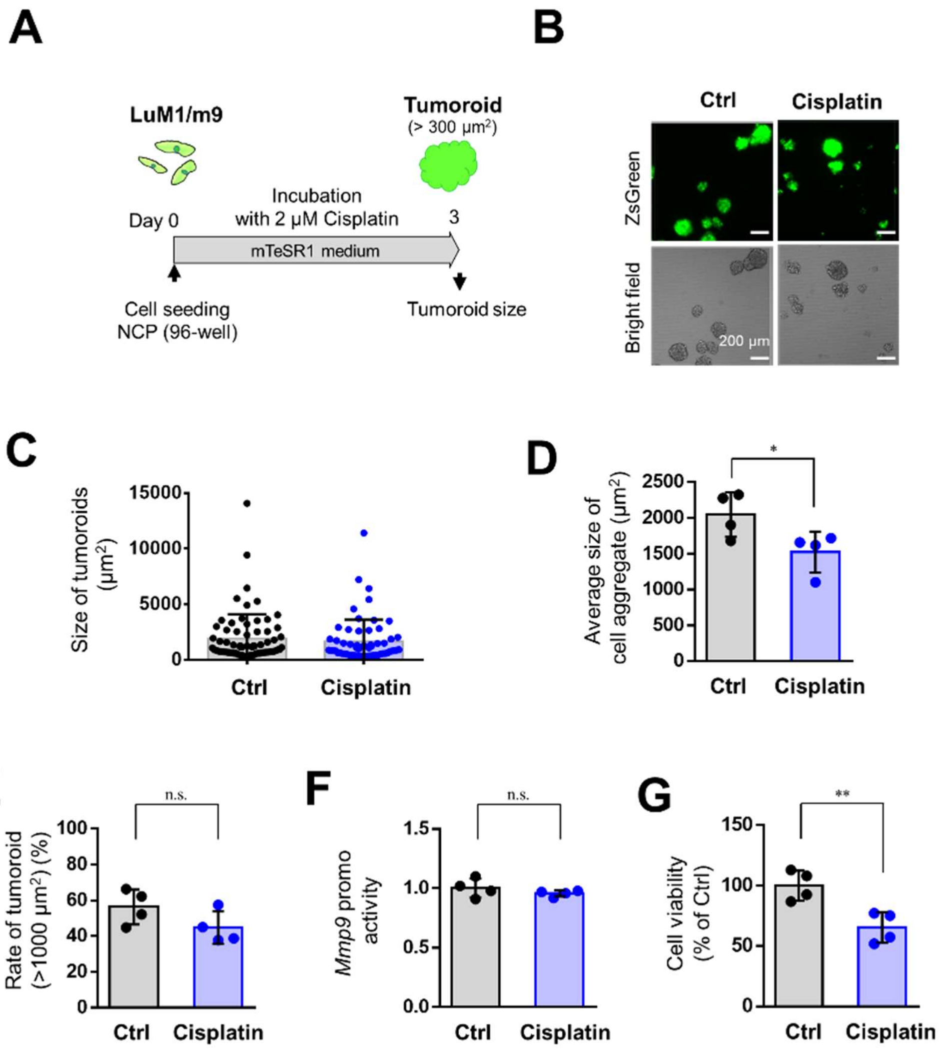

Figure S1. Effects of Cisplatin (at 2 μM) on tumoroid formation in mTeSR1 medium. (A) A scheme of the experimental protocol used for panel B-G. LuM1/m9 cells were cultured with 2 μM Cisplatin for 3 days in the mTeSR1 medium. (B) Representative images of cell aggregates. Scale bars, 200 μm. (C) Column scatters plotting of tumoroid size. A representative data from a single well were shown. (D) Average tumoroid size. n= 4 independent culture wells. (E) The rate of tumoroids larger than 1,000 μm². n= 4 independent culture wells. (F) *Mmp9* promoter activity. n= 4 independent culture wells. (G) Cell viability. n=4 independent culture wells. *P<0.05, **P<0.01, n.s., not significant.

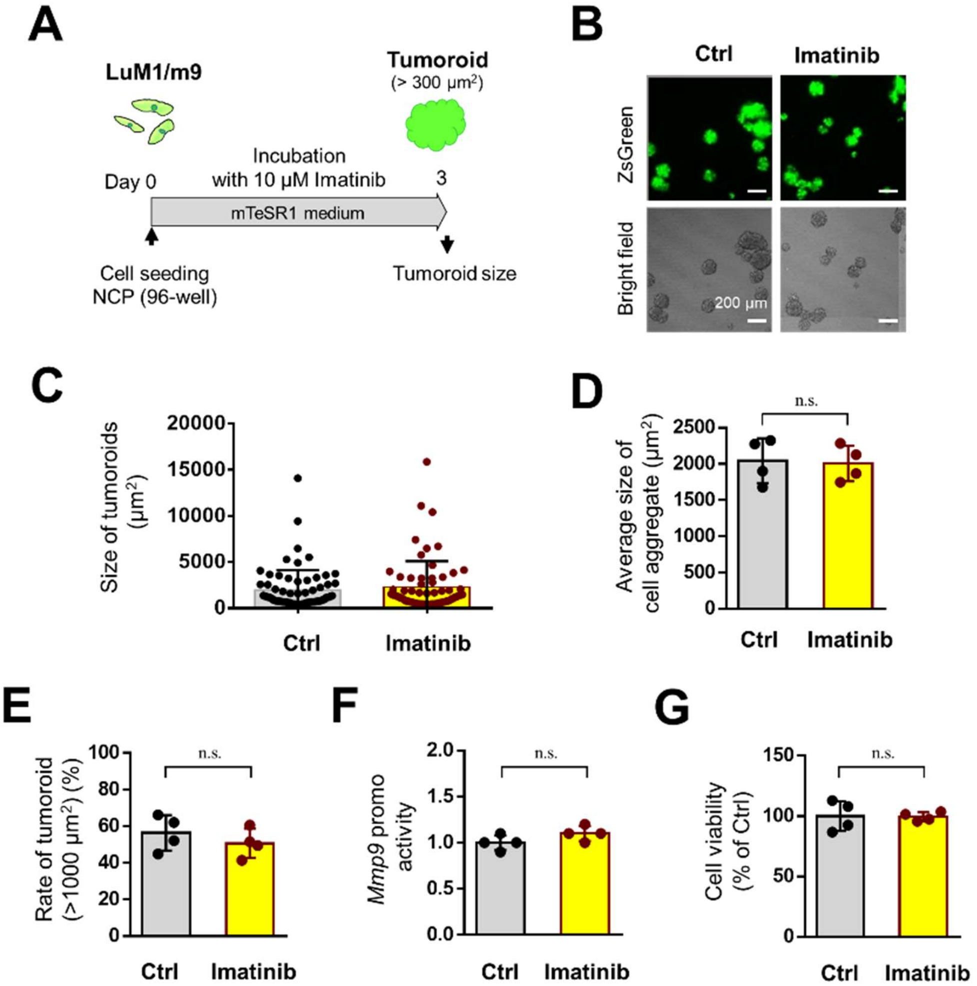

Figure S2. Imatinib (at 10 μM) did not alter tumoroid formation in mTeSR1 medium. (A) A scheme of the experimental protocol used for panel B-G. LuM1/m9 cells were cultured with 10 μM Imatinib for 3 days in the mTeSR1 medium. (B) Representative images of cell aggregates. Scale bars, 200 μm. (C) Column scatters plotting of tumoroid size. A representative data from a single well were shown. (D) Average tumoroid size. n= 4 independent culture wells. (E) The rate of tumoroids larger than 1,000 μm². n= 4 independent culture wells. (F) *Mmp9* promoter activity. n= 4 independent culture wells. (G) Cell viability. n=4 independent culture wells. n.s., not significant.

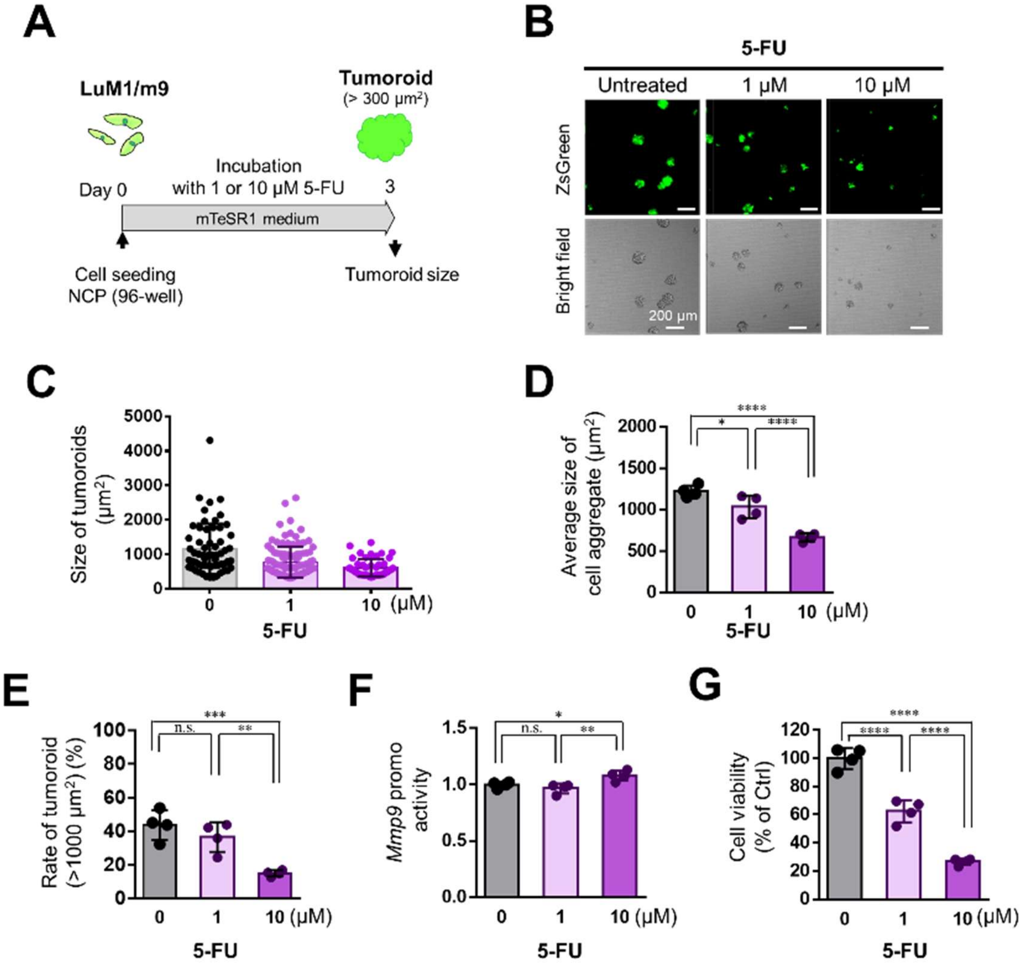

Figure S3. 5-FU inhibited tumoroid growth of mCRC in mTeSR1 medium, although not completely. (A) A scheme of the experimental protocol used for panel B-G. LuM1/m9 cells were cultured with 5-FU at 0, 1, or 10 μM for 3 days in the mTeSR1 medium. (B) Representative images of cell aggregates. Scale bars, 200 μm. (C) Column scatters plotting of tumoroid size. A representative data from a single well were shown. (D) Average tumoroid size. n= 4 independent culture wells. (E) The rate of tumoroids larger than 1,000 μm². n= 4 independent culture wells. (F) *Mmp9* promoter activity. n= 4 independent culture wells. (G) Cell viability. n=4 independent culture wells. *P<0.05, **P<0.01, ***P<0.001, ****P<0.0001, n.s., not significant.

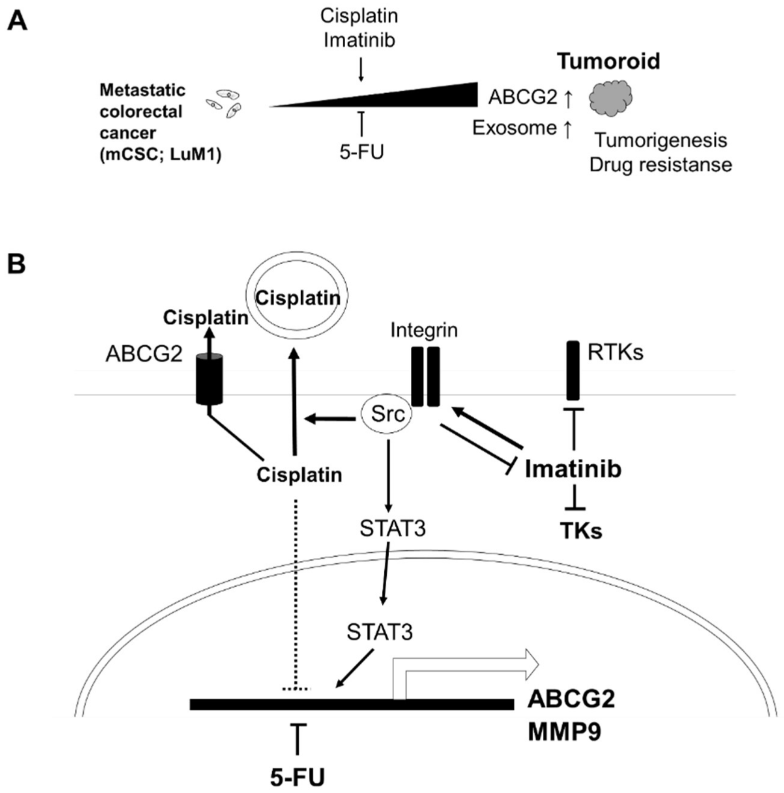

Figure S4. Data interpretation and perspective. (A) Cisplatin and Imatinib promoted tumoroid growth in mCRC cells, whereas 5-FU inhibited tumoroid growth, although not completely. Tumoroid growth is accompanied by the expression of ABCG2, a drug efflux pump, and the release of extracellular vesicles such as exosomes. (B) Potential mechanisms of chemoresistance to Cisplatin and Imatinib. Cisplatin can be secreted via drug efflux pumps such as ABCG2 and/or with exosomes. Imatinib can activate Integrin-Src-STAT3 signaling that may promote cellular survival.
